# The association of *ROS1* mutation with cancer immunity and its impact on the efficacy of pan-cancer immunotherapy

**DOI:** 10.1186/s12967-024-05166-y

**Published:** 2024-04-30

**Authors:** Yingying Li, Hong Zhao, Jinyuan Huang, Huimeng Yan, Bin Zhao

**Affiliations:** 1 Quanzhou First Hospital Affiliated to Fujian Medical University, Quanzhou, China; 2 Second Affiliated Hospital, Yuying Children’s Hospital, Wenzhou Medical University, Wenzhou, China; 3 The Cancer Center of The Fifth Affiliated Hospital of Sun Yat-Sen University, Zhuhai, China

**Keywords:** *ROS1*, Immunotherapy, Biomarker, Tumor immunogenicity, cancer

To the editor,

In the past decade, immunotherapy has revolutionized cancer treatment. However, it is still difficult to determine which patients should be offered immune checkpoint inhibitors (ICIs) currently [1]. Mutations of *ROS1* play important roles in cell activation, differentiation, proliferation and survival [2], which may affect the tumor immunogenicity. Indeed, evidences from both *in vivo* and *in vitro* studies revealed *ROS1* could regulate the expression of *PD-L1* and participate in immune escape through the activation of *ROS1-SHP2*- and *MEK-ERK*-signaling pathways in lung cancer [2]. In melanoma, *ROS1* mutation was associated with an enrichment of DNA-damage-response-related processes and DNA-repair-related molecules [3], which might lead to the enhanced immune surveillance. Here, we conducted a comprehensive bioinformatic and clinical analysis to study the characteristics of *ROS1* mutation and its association with pan-cancer immunotherapy (Suppl. Method).

First, 1610 patients with 10 tumor types were applied as a discovery cohort (Table S1), *ROS1* mutation was associated with longer overall survival (OS, hazard ratio [HR] = 0.53; 95% CI, 0.41–0.68; *P* < 0.001; Fig. 1A). We then collected 1395 patients with 7 tumor types from 9 datasets as a validation cohort (Table S1). 146 patients with *ROS1*-mutant tumors achieved favorable OS (HR = 0.74; 95% CI, 0.59–0.92; *P* = 0.01; Fig. 1B). Totally, in 3005 patients with 12 tumor types, *ROS1* mutation decreased the risk of death by 37% (HR = 0.63; 95% CI, 0.54–0.74; *P* < 0.001; Fig. 1C). Additionally, more patients with *ROS1*-mutant tumors responded to ICIs (35.3% vs. 22.5%; *P* < 0.001; Fig. 1D). Further univariate (Fig. 1E) and multivariate (Fig. 1F) Cox analysis demonstrated *ROS1* mutation was an independent predictor. Thereby, we constructed a nomogram to estimate the 12-month and 24-month survival after the initiation of immunotherapy (Fig. 1G). As shown in Fig. 1H, the performance of this cure-model-based nomogram was good. Moreover, we classified patients into low- and high-score subgroups based on the optimal cutoff value estimated by X-tile software. Low-score was associated with better OS in both discovery cohort (HR = 0.38; 95% CI, 0.32–0.46; *P* < 0.001; Fig. 1I) and validation cohort (HR = 0.71; 95% CI, 0.57–0.86; *P* = 0.003; Fig. 1J).

**Fig. 1:**
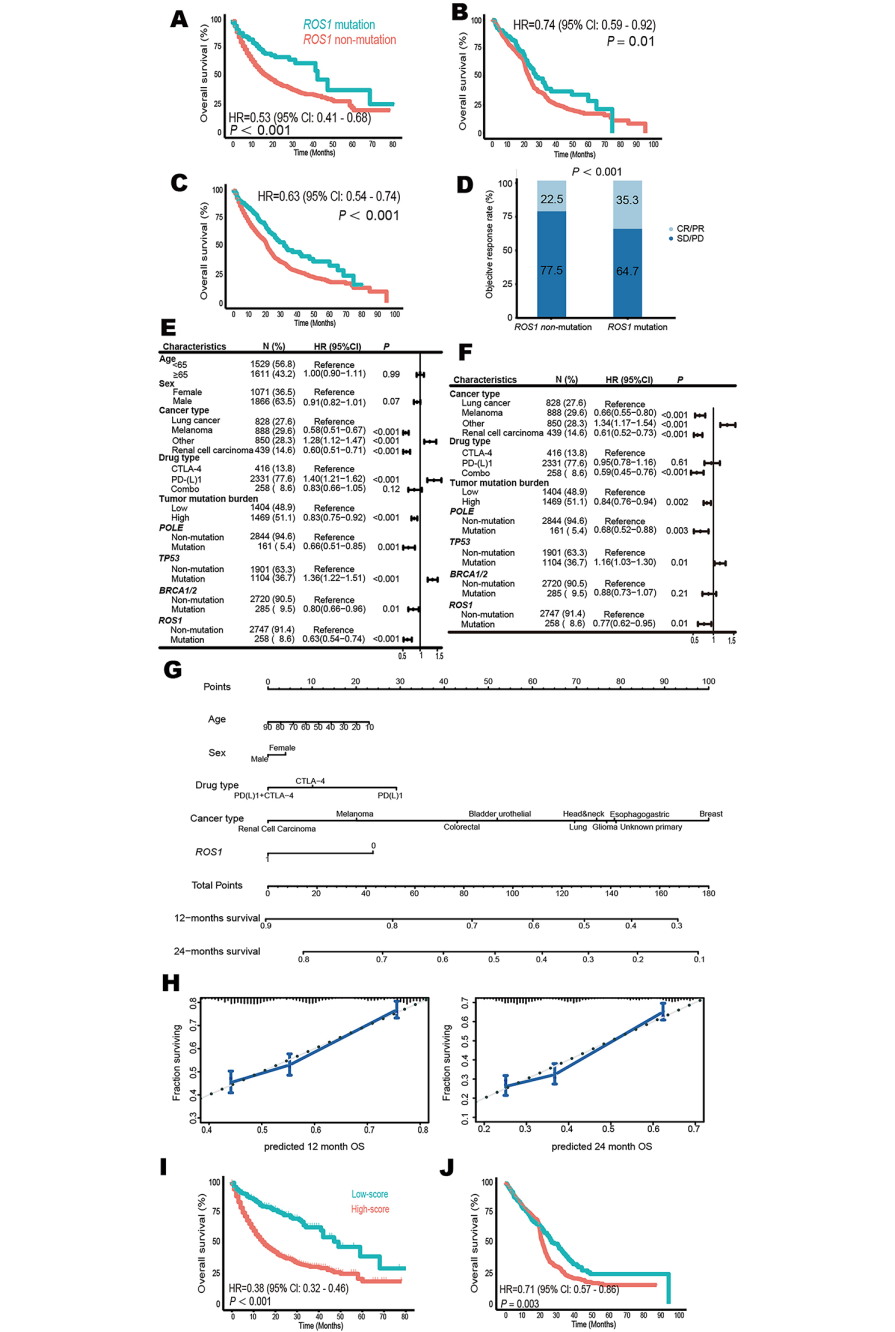
*ROS1* mutation is an independent biomarker for favorable outcomes in pan-cancer immunotherapy. (**A**) Kaplan–Meier survival analysis stratified by *ROS1* mutation status in 1610 cancer patients with 10 tumor types treated with ICIs in the discovery cohort. (**B**) Association between *ROS1* mutation and OS in 1395 patients with 7 tumor types in the validation cohort. (**C-D**) Comparison of OS (C) and ORR (D) between patients with *ROS1* mutant tumors and patients with *ROS1* non-mutant tumors in 3005 subjects with 12 tumors treated with ICIs. (**E-F**) Univariate (E) and multivariate (F) Cox analysis of the association between *ROS1* mutation and OS in 3888 patients with 12 tumors treated with ICIs. (**G**) Nomogram to predict the 12- and 24-month survival. (**H**) Calibration plots for validation of the 12- and 24-month survival from the nomogram in the discovery cohort. The average predicted probability (X axis) was plotted against the observed Kaplan-Meier estimate in the subgroup (Y axis, 95% CIs of the estimates are presented as vertical lines). Continuous line is the reference line, indicating what an optimal nomogram would be. (**I-J**) Based on the optimal cutoff value derived from nomogram, low-score was associated with favorable OS in both discovery cohort (I) and validation cohort (J). CI, confidence interval; CR, complete response; HR, hazard ratio; ICI, immune checkpoint inhibitor; ORR, objective response rate; OS, overall survival; PD, progressive disease; PR, partial response; SD, stable disease

To investigate the underlying mechanisms between cancer immunotherapy and *ROS1* mutation, multi-omics information extracted from The Cancer Genome Atlas (TCGA) were explored. Totally, 460 of 10,953 patients (4.20%) harbored *ROS1* mutations (Figure S1A). Of all the identified 665 mutations, 81.35% were missense, 12.78% were truncating, 4.21% were spice, and 1.65% were fusion mutations. These mutations occurred in a dispersed manner throughout the whole sequence (Figure S1B). Further analysis revealed *ROS1* mutations were independent of disease-free survival, disease-specific survival, progression-free survival, and OS (Figure S1C).

We compared the key intrinsic immune features in *ROS1*-mutant and *ROS1*-non-mutant tumors. The mutation loads including tumor mutation burden (TMB), silent mutation rate, and non-silent mutation rate were significantly upregulated in *ROS1*-mutant tumors (Fig. 2A). Next, we examined the association between mutant signatures and OS in *ROS1*-mutant patients (Figure S2). The frequencies of SBS7a, SBS10b, SBS16, and SBS32 were significantly different between *ROS1*-mutant and *ROS1*-non-mutant tumors. These SBSs were further identified as robust biomarkers for OS in pan-cancer immunotherapy. Additionally, the expression levels of *PD-1, PD-L1* and *CTLA-4* were significantly higher in *ROS1*-mutant tumors (Fig. 2B). *ROS1* mutation was associated with increased MHC-related antigen-presenting molecules and co-stimulators (Fig. 2G).

**Fig. 2:**
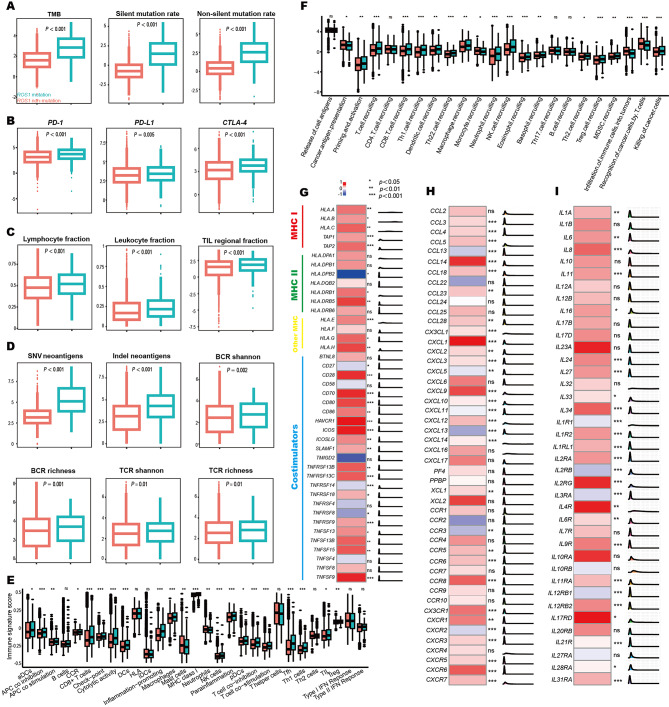
The tumor immune microenvironment in patients with*ROS1-* mutant and *ROS1*-non-mutant tumors. (**A**) Comparison of TMB, silent mutation rate, and non-silent mutation rate between *ROS1-*mutant and *ROS1*-non-mutant tumors. (**B**) mRNA expression levels of *PD-1, PD-L1*, and *CTLA-4*. (**C**) The immune cell infiltration revealed by leukocyte fractions, lymphocytes fraction and tumor-infiltrating lymphocyte fraction. (**D**) The abundances of SNV neoantigens/Indel neoantigens and the diversity of TCR/BCR. (**E**) Differences of 29 immune signatures estimated by ssGSEA. (**F**) Comparison of 23 immune signatures measured by the cancer immunity cycle. (**G**) Expression differences of 16 MHC-related antigen-presenting molecules and 25 co-stimulators. (**H**) Comparison of 48 chemokines and their receptors between *ROS1-*mutant and *ROS1-*non-mutant tumors. (**I**) Expression of 39 immune-stimulators in *ROS1-*mutant and *ROS1-*non-mutant tumors. BCR, B cell receptor; CTLA-4, cytotoxic T-lymphocyte-associated antigen 4; MHC, major histocompatibility complex; PD-1, programmed cell death protein 1; PD-L1, programmed cell death ligand 1; SNV, single nucleotide variants; TCR, T cell receptor; TIL, tumor-infiltrating lymphocyte; TMB, tumor mutation burden

The major extrinsic immune characteristics were also examined here. *ROS1* mutation was associated with higher levels of immune cell infiltration based on lymphocytes fraction, leukocyte fractions, and tumor-infiltrating lymphocyte fraction (Fig. 2C). Mutations may induce potential tumor-associated neoantigens, which are recognized by T cells with T cell receptors (TCRs) or B cells with B cell receptors (BCRs). The abundances of SNV/Indel neoantigens and the diversity of TCR/BCR were significantly upregulated in *ROS1*-mutant tumors (Fig. 2D). ssGSEA presented 29 key immune pathways, cells, and functions [4]. The cancer immunity cycle reflected the functions of immunomodulators and chemokines by estimate cancer antigen presentation, the release of antigens, priming and activation, immune cell recruitment and infiltration, recognition and killing of tumor cells [5]. As shown in Fig. 2E and Fig. 2F, the immune cell populations and immune activities were clearly enriched in *ROS1*-mutant tumors. Of note, the abundances of CD8^+^ T cells, which were critical for cancer immunity, were significantly increased in *ROS1*-mutant tumors. Moreover, we examined the expression levels of 48 chemokines and their receptors (Fig. 2H) and 39 immune-stimulators (Fig. 2I), most of which were elevated in *ROS1*-mutant tumors.

In summary, these results revealed *ROS1* mutation was a favorable biomarker for outcomes in pan-cancer immunotherapy.

### Electronic supplementary material

Below is the link to the electronic supplementary material.

**Suppl. Figure 1**. The characteristics of ROS1 mutation in 33 tumor types based on TCGA cohort. (A) The mutant frequencies of ROS1 gene across 33 tumor types. (B) The subtypes and distributions of ROS1 somatic mutations. X-axis, amino acid; Y-axis, numbers of ROS1 mutations. fn3, Fibronectin type III domain (102-169; 202? 274; 1061-1138; 1668-1738); Pkinase_Tyr; Protein tyrosine kinase (1947 - 2215). Green, missense mutation; black, truncating mutation; orange, spice mutation; purple, fusion mutation. (C) Comparison of DFS, DSS, PFS and OS between patients with ROS1 mutation and patients with ROS1 non-mutation in 10953 subjects with 33 tumor types. DFS, disease-free survival; DSS, disease-specific survival; HR, hazard ratio; PFS, progression-free survival; OS, overall survival.

**Suppl Figure 2**. COSMIC reference signatures associated with ROS1 mutation. (A) The illustrations of four identified SBS signatures related with ROS1 mutation and their frequencies in ROS1-mutant and ROS1-non-mutant tumors. Bold black, SBS signature and its known etiologies. Green, frequency in ROS1-mutant cancer. Orange, frequency in ROS1-non-mutant cancer. (B) The associations between four identified mutation signatures with OS in cancer immunotherapy.HR, hazard ratio; OS, overall survival; SBS, Single base substitution.

Supplementary Material 3

Supplementary Material 4

## Data Availability

The datasets generated during and/or analyzed during the current study are available from the corresponding author upon reasonable request.
