# Supplementary figures and images for: The association of ROS1 mutation with cancer immunity and its impact on the efficacy of pan-cancer immunotherapy
Source: J Transl Med. 2024 Apr 30;22:403. doi: 10.1186/s12967-024-05166-y (PMC11061941; doi:10.1186/s12967-024-05166-y)

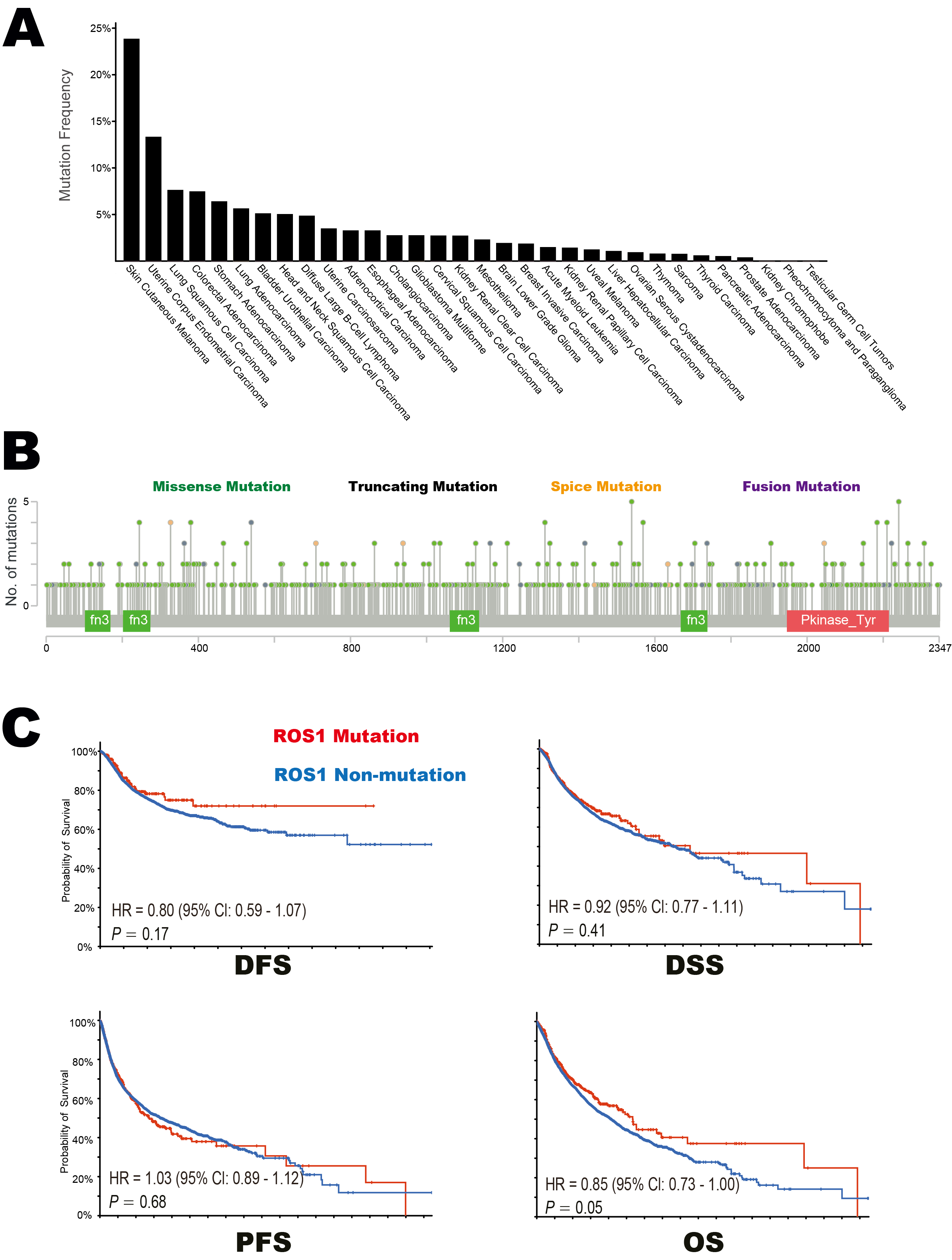

Supplement: Supplementary file 1 — Suppl. Figure 1. The characteristics of ROS1 mutation in 33 tumor types based on TCGA cohort. (A) The mutant frequencies of ROS1 gene across 33 tumor types. (B) The subtypes and distributions of ROS1 somatic mutations. X-axis, amino acid; Y-axis, numbers of ROS1 mutations. fn3, Fibronectin type III domain (102-169; 202? 274; 1061-1138; 1668-1738); Pkinase_Tyr; Protein tyrosine kinase (1947 - 2215). Green, missense mutation; black, truncating mutation; orange, spice mutation; purple, fusion mutation. (C) Comparison of DFS, DSS, PFS and OS between patients with ROS1 mutation and patients with ROS1 non-mutation in 10953 subjects with 33 tumor types. DFS, disease-free survival; DSS, disease-specific survival; HR, hazard ratio; PFS, progression-free survival; OS, overall survival. [file 12967_2024_5166_MOESM1_ESM.jpg]

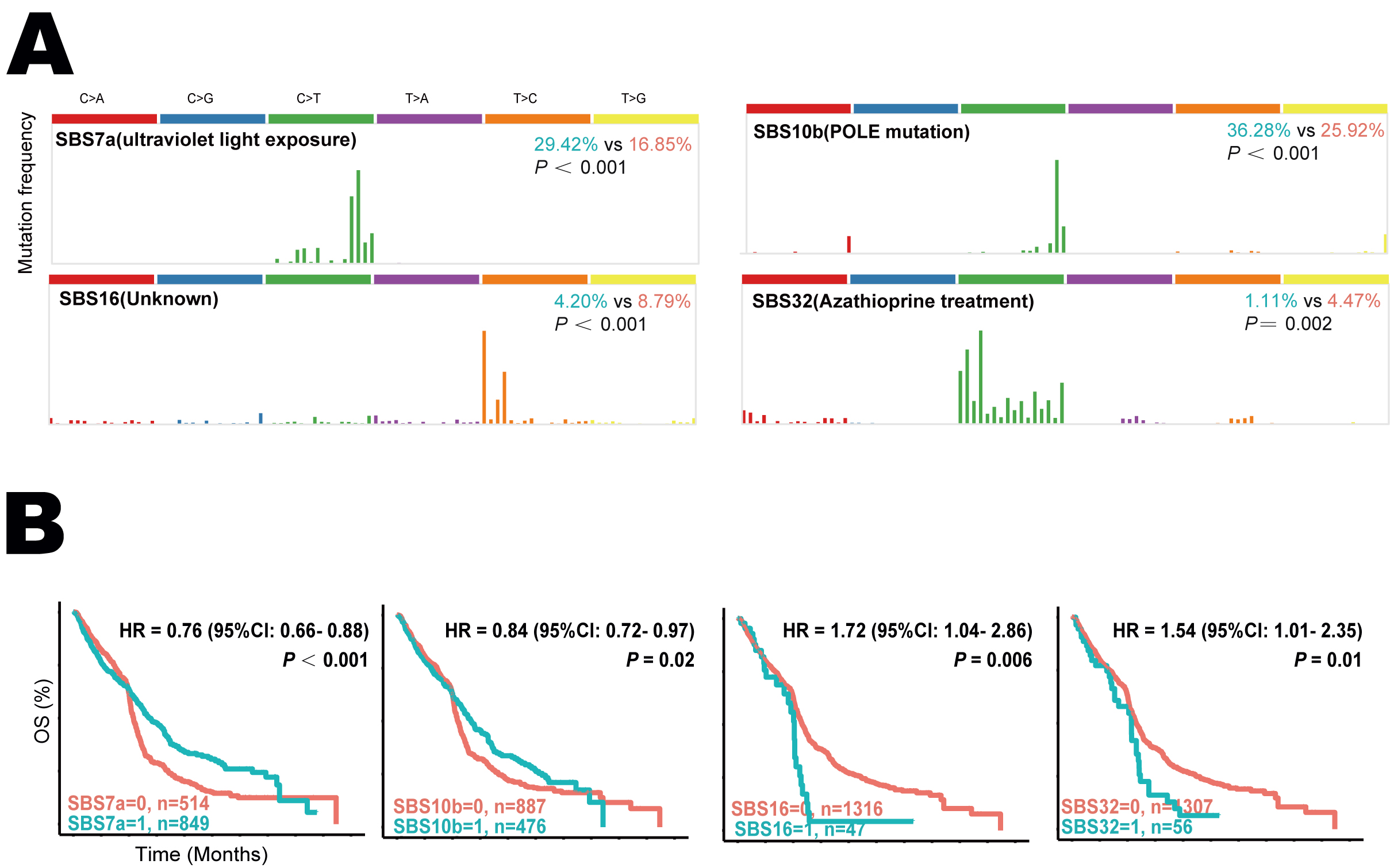

Supplement: Supplementary file 2 — Suppl Figure 2. COSMIC reference signatures associated with ROS1 mutation. (A) The illustrations of four identified SBS signatures related with ROS1 mutation and their frequencies in ROS1-mutant and ROS1-non-mutant tumors. Bold black, SBS signature and its known etiologies. Green, frequency in ROS1-mutant cancer. Orange, frequency in ROS1-non-mutant cancer. (B) The associations between four identified mutation signatures with OS in cancer immunotherapy.HR, hazard ratio; OS, overall survival; SBS, Single base substitution. [file 12967_2024_5166_MOESM2_ESM.jpg]
