# Supplementary material for: The association of ROS1 mutation with cancer immunity and its impact on the efficacy of pan-cancer immunotherapy
Source: J Transl Med. 2024 Apr 30;22:403. doi: 10.1186/s12967-024-05166-y (PMC11061941; doi:10.1186/s12967-024-05166-y)
Supplement: Supplementary file 3 — Supplementary Material 3 [file 12967_2024_5166_MOESM3_ESM.docx]

**Suppl. Table 1.** Baseline characteristics of the trials included in the immunotherapy analysis.

| **Author, year** | **Treatment agents** | **Cancer type** | **Detection method** | ***ROS1* mutation** | **N** | **No. of responses** |
| --- | --- | --- | --- | --- | --- | --- |
| **Discovery cohort** | | | | | | |
| Samstein, 2019 ^1^ | Inhibitors targeting CTLA-4, PD-1, and PD-L1 | Multiple tumors | MSK-IMPACT panel | Positive | 112 | 2 |
|  |  |  |  | Negative | 1498 | 50 |
| **Validation cohort** | | | | | | |
| Hugo, 2016 ^2^ | Pembrolizumab/Nivolumab | Melanoma | WES | Positive | 11 | 6 |
|  |  |  |  | Negative | 26 | 14 |
| Liu, 2019 ^3^ | Pembrolizumab/Nivolumab | Melanoma | WES | Positive | 28 | 14 |
|  |  |  |  | Negative | 116 | 41 |
| Miao, 2018 ^4^ | Inhibitors targeting CTLA-4, PD-1, and PD-L1 | Multiple tumors | WES | Positive | 38 | 11 |
|  |  |  |  | Negative | 211 | 59 |
| Riaz, 2017 ^5^ | Nivolumab | Melanoma | WES | Positive | 11 | 6 |
|  |  |  |  | Negative | 57 | 9 |
| Van Allen, 2015 ^6^ | Ipilimumab | Melanoma | WES | Positive | 20 | 4 |
|  |  |  |  | Negative | 90 | 13 |
| Gandara, 2018 ^7^ | Atezolizumab | Lung cancer | WES | Positive | 21 | 5 |
|  |  |  |  | Negative | 406 | 57 |
| Snyder, 2014 ^8^ | Ipilimumab/Tremelimumab | Melanoma | WES | Positive | 9 | NA |
|  |  |  |  | Negative | 55 | NA |
| Miao, 2018 ^9^ | Nivolumab | Renal cancer | WES | Positive | 1 | 0 |
|  |  |  |  | Negative | 34 | 7 |
| Braun, 2020^10^ | Nivolumab | Renal cancer | WES | Positive | 7 | 1 |
|  |  |  |  | Negative | 254 | 55 |

NA, not available; WES, whole-exome sequencing

**References:**

1. Samstein RM, Lee CH, Shoushtari AN, et al. Tumor mutational load predicts survival after immunotherapy across multiple cancer types. *Nat Genet* 2019; **51**(2): 202-6.

2. Hugo W, Zaretsky JM, Sun L, et al. Genomic and Transcriptomic Features of Response to Anti-PD-1 Therapy in Metastatic Melanoma. *Cell* 2016; **165**(1): 35-44.

3. Liu D, Schilling B, Liu D, et al. Integrative molecular and clinical modeling of clinical outcomes to PD1 blockade in patients with metastatic melanoma. *Nature medicine* 2019; **25**(12): 1916-27.

4. Miao D, Margolis CA, Vokes NI, et al. Genomic correlates of response to immune checkpoint blockade in microsatellite-stable solid tumors. *Nat Genet* 2018; **50**(9): 1271-81.

5. Riaz N, Havel JJ, Makarov V, et al. Tumor and Microenvironment Evolution during Immunotherapy with Nivolumab. *Cell* 2017; **171**(4): 934-49 e16.

6. Van Allen EM, Miao D, Schilling B, et al. Genomic correlates of response to CTLA-4 blockade in metastatic melanoma. *Science (New York, NY)* 2015; **350**(6257): 207-11.

7. Gandara DR, Paul SM, Kowanetz M, et al. Blood-based tumor mutational burden as a predictor of clinical benefit in non-small-cell lung cancer patients treated with atezolizumab. *Nature medicine* 2018; **24**(9): 1441-8.

8. Snyder A, Makarov V, Merghoub T, et al. Genetic basis for clinical response to CTLA-4 blockade in melanoma. *N Engl J Med* 2014; **371**(23): 2189-99.

9. Miao D, Margolis CA, Gao W, et al. Genomic correlates of response to immune checkpoint therapies in clear cell renal cell carcinoma. *Science* 2018; **359**(6377): 801-6.

10. Braun DA, Hou Y, Bakouny Z, et al. Interplay of somatic alterations and immune infiltration modulates response to PD-1 blockade in advanced clear cell renal cell carcinoma. *Nature medicine* 2020; **26**(6): 909-18.
