# Supplementary material for: The association of ROS1 mutation with cancer immunity and its impact on the efficacy of pan-cancer immunotherapy
Source: J Transl Med. 2024 Apr 30;22:403. doi: 10.1186/s12967-024-05166-y (PMC11061941; doi:10.1186/s12967-024-05166-y)
Supplement: Supplementary file 4 — Supplementary Material 4 [file 12967_2024_5166_MOESM4_ESM.docx]

**Suppl. Methods**

**Data collection for *ROS1*-mutant analysis**

*ROS1* mutation status in patients treated with ICIs were extracted from 10 previously published manuscripts[1] [2] [3] [4] [5] [6] [7] [8] [9, 10]. The baseline features of the eligible studies were shown in Suppl. Table 1. Among them, lung cancer was investigated in 3 studies with 828 patients [3] [10] [6], melanoma in 7 studies with 888 patients [1] [2, 3] [4, 10] [5, 7] , renal cell carcinoma in 4 studies with 439 patients [3] [10] [8, 9], bladder urothelial cancer in 2 studies with 238 patients [3] [10], head and neck cancer in 2 studies with 141 patients [3] [10], esophagogastric cancer in 2 studies with 118 patients [3] [10], glioma in 1 study with 116 patients[10], colorectal cancer in 1 study with 109 patients[10], cancer of unknown primary in 1 study with 85 patients[10], breast cancer in 1 study with 41 patients[10], sarcoma in 1 study with 41 patients[10], and anal cancer in 1 study with 41 patients[10]. Inhibitors targeting CTLA-4 were administrated in 4 studies[3, 5, 7, 10], targeting PD-1/PD-L1 in 8 cohorts [1-4, 6, 8-10]. Additionally, whole-exome sequencing (WES) was employed for sequencing in 9 cohorts [1] [2] [3] [4] [5] [6] [7] [8] [9]. MSK-IMPACT panel was applied in 1 study with multiple tumors [10].

Overall survival (OS) referred to the time from the date of first immunotherapy to the time of death or last follow-up. Response Evaluation Criteria in Solid Tumors (RECIST) version 1.1 was applied to estimate the objective response rate (ORR). Patients who showed complete response (CR) or partial response (PR) were categorized as responders; patients who experienced stable disease (SD) or progressive disease (PD) were classified as non-responders.

Information regarding RNA expression, DNA methylation and sequencing, and clinicopathological characteristics of 33 tumor types were obtained from the Cancer Genome Atlas (TCGA, https://gdc.cancer.gov). Key features related to tumor immune micro-environment (silent mutation rate, non-silent mutation rate, SNV neoantigen, indel neoantigen, lymphocyte fraction, leukocyte fraction, CD8 T cell abundance, TIL regional fraction, TCR richness, and TCR Shannon index) were calculated as previously described [11].

The ‘deconstructSigs’ R package was applied to perform non-negative matrix factorization analysis of mutations. The extracted mutation pattern was compared against the COSMIC based on cosine similarity.

Nomogram is widely used to predict outcomes quantitatively in oncology using critical predictive features. A calibration curve can be used to evaluate the similarity between the predictive and actual survival probability. The ‘rms’ R package was used to generate both nomogram and calibration curves.

**Statistics**

Survival curves were generated by Kaplan-Meier method to reflect the differences in survival and the log-rank test was used to evaluate the statistical significance of differences. Wilcoxon test, Kruskal-Wallis, and Chi-square test was used to analyze the associations among various categorical variables depend on the context. Hazard ratio (HR) was calculated by Cox proportional hazards model and 95% CI was reported. mPFS, mOS and their 95% CIs were presented where relevant. All data processing and analysis were performed with R software (version 4.2.1). Two-sided *P* <0.05 were considered statistically significant.

**References:**

1. Hugo, W., et al., *Genomic and Transcriptomic Features of Response to Anti-PD-1 Therapy in Metastatic Melanoma.* Cell, 2016. **165**(1): p. 35-44.

2. Liu, D., et al., *Integrative molecular and clinical modeling of clinical outcomes to PD1 blockade in patients with metastatic melanoma.* Nat Med, 2019. **25**(12): p. 1916-1927.

3. Miao, D., et al., *Genomic correlates of response to immune checkpoint blockade in microsatellite-stable solid tumors.* Nat Genet, 2018. **50**(9): p. 1271-1281.

4. Riaz, N., et al., *Tumor and Microenvironment Evolution during Immunotherapy with Nivolumab.* Cell, 2017. **171**(4): p. 934-949 e16.

5. Van Allen, E.M., et al., *Genomic correlates of response to CTLA-4 blockade in metastatic melanoma.* Science, 2015. **350**(6257): p. 207-211.

6. Gandara, D.R., et al., *Blood-based tumor mutational burden as a predictor of clinical benefit in non-small-cell lung cancer patients treated with atezolizumab.* Nat Med, 2018. **24**(9): p. 1441-1448.

7. Snyder, A., et al., *Genetic basis for clinical response to CTLA-4 blockade in melanoma.* N Engl J Med, 2014. **371**(23): p. 2189-2199.

8. Miao, D., et al., *Genomic correlates of response to immune checkpoint therapies in clear cell renal cell carcinoma.* Science, 2018. **359**(6377): p. 801-806.

9. Braun, D.A., et al., *Interplay of somatic alterations and immune infiltration modulates response to PD-1 blockade in advanced clear cell renal cell carcinoma.* Nat Med, 2020. **26**(6): p. 909-918.

10. Samstein, R.M., et al., *Tumor mutational load predicts survival after immunotherapy across multiple cancer types.* Nat Genet, 2019. **51**(2): p. 202-206.

11. Thorsson, V., et al., *The Immune Landscape of Cancer.* Immunity, 2018. **48**(4): p. 812-830.e14.
